# Investigating Osteomyelitis as a Rare Adverse Effect of Vaccination in the Pediatric Population

**DOI:** 10.3390/pathogens13110972

**Published:** 2024-11-07

**Authors:** Valeria Garbo, Laura Venuti, Chiara Albano, Costanza Caruana, Alessandra Cuccia, Anna Condemi, Giovanni Boncori, Valentina Frasca Polara, Antonio Cascio, Sergio Salerno, Claudia Colomba

**Affiliations:** 1 Department of Health Promotion, Mother and Child Care, Internal Medicine and Medical Specialties “G D’Alessandro”, University of Palermo, 90127 Palermo, Italy; valeria.garbo@arnascivico.it (V.G.);; 2 Division of Paediatric Infectious Disease, “G. Di Cristina” Hospital, ARNAS Civico Di Cristina Benfratelli, 90127 Palermo, Italy; 3 Infectious and Tropical Disease Unit, Sicilian Regional Reference Center for the Fight Against AIDS, AOU Policlinico “P. Giaccone”, 90127 Palermo, Italy; 4 Department of Biomedicine, Neuroscience and Advanced Diagnostics, University of Palermo, 90127 Palermo, Italy

**Keywords:** pediatric osteomyelitis, vaccination adverse effects, vaccine-associated infections, *Staphylococcus aureus*, immunization procedures, skin preparation, needle length, vaccine adverse event reporting system (VAERS), injection site infections, vaccine safety

## Abstract

Immunization is a preventive measure of crucial importance. As with any other medication, side effects are a possibility and include the rare occurrence of severe infections, such as osteomyelitis. We report an unusual case of pediatric osteomyelitis following vaccination and provide a review of similar reports submitted to the Vaccine Adverse Event Report System (VAERS), aiming to explore the association between the vaccination procedure and the occurrence of osteomyelitis in childhood. A previously healthy infant, with no history of trauma or infection, presented with hyperpyrexia, swelling, and functional impairment in the left leg and was eventually diagnosed with osteomyelitis of the left femur. An edema was noted at the site of the injection that he received days before for immunization purposes. The infection required surgical drainage and a four-week-long intravenous antibiotic treatment, and the patient was discharged upon showing improved clinical conditions. Forty-seven reports of similar cases submitted to VAERS between 1994 and 2023 were collected, and several cases from the literature, including a case of femoral osteomyelitis in a newborn vaccinated against Hepatitis B, attributed to improper injection technique. Another case was reported in a 15-year-old girl, which aligned with six similar cases of osteomyelitis in adolescents following HPV vaccines collected from VAERS. Despite the small sample number, the findings that in 77% of cases the infection was localized in the vaccinated limb and that symptoms appeared on average 4.3 days (IQR 1.0–5.7 days) post-vaccination suggest a possible link to the injection procedure and highlight the need to adhere to recommendations regarding skin preparation and the selection of the appropriate needle length and injection site.

## 1. Background

Vaccinations are the most effective and safe intervention for preventing major infectious diseases and represent an essential component of global public health programs. Over the last millennium, they have contributed to eradicating or reducing the burden of many diseases, saving more human lives than any other medical procedure in history [1].

The surveillance of adverse effects following immunization is mandatory. In Italy, data are collected by a national pharmacovigilance network known as “Rete Nazionale di Farmacovigilanza (RNF)”, which includes the Italian Medicine Agency (Agenzia Italiana del Farmaco, AIFA), the 20 regions and the autonomous provinces of Trento and Bolzano, 204 local health authorities, 112 hospitals, 38 research institutes, and 561 pharmaceutical companies [2,3].

The most common side effects of vaccination are warmth, swelling, pain at the injection site, fever, headache, and fatigue [4]. Allergic reactions can also occur; therefore, a 15–30 min long observation period is recommended post-procedure [4,5]. Bacterial infections following vaccination, however rare, have been occasionally reported and include cellulitis [6,7], abscesses [8,9,10,11], septic arthritis [12,13,14,15], bacteremia [16], and osteomyelitis [17,18,19]. In the United States, between 1991 and 2001, more than 1.9 billion doses of vaccines were distributed and 128,717 adverse events were reported to the Vaccine Adverse Event Report System (VAERS) [20]. Of these, 2148 (1.7%) were reports of cellulitis and 426 (0.3%) were reports of abscesses at the injection site [20].

Common responsible agents for these infections are Gram-positive cocci, especially *Staphylococcus aureus* (*S. aureus*), an opportunistic commensal residing in the nostrils, oropharynx, and skin of healthy individuals [21]. *S. aureus* can cause a broad spectrum of conditions, including skin and soft tissue infections, osteomyelitis, pneumonia, bacteremia, endocarditis, sepsis, and toxic shock syndrome (TSS) [22].

We describe a case of osteomyelitis in a previously healthy infant, likely caused by the direct inoculation of the microorganism occurring during the vaccination procedure and provide a review of pediatric cases of osteomyelitis following vaccination collected from VAERS, with the objective of exploring the association between the vaccination procedure and the occurrence of osteomyelitis in childhood.

## 2. Case Report

A 3-month-old previously healthy infant was admitted to our pediatric infectious disease department in November 2023 because of swelling at the site of injection in the left lower limb, functional impairment, and hyperpyrexia. He had been immunized against meningococcus B and rotavirus about 12 days before admission.

**Figure 1:**
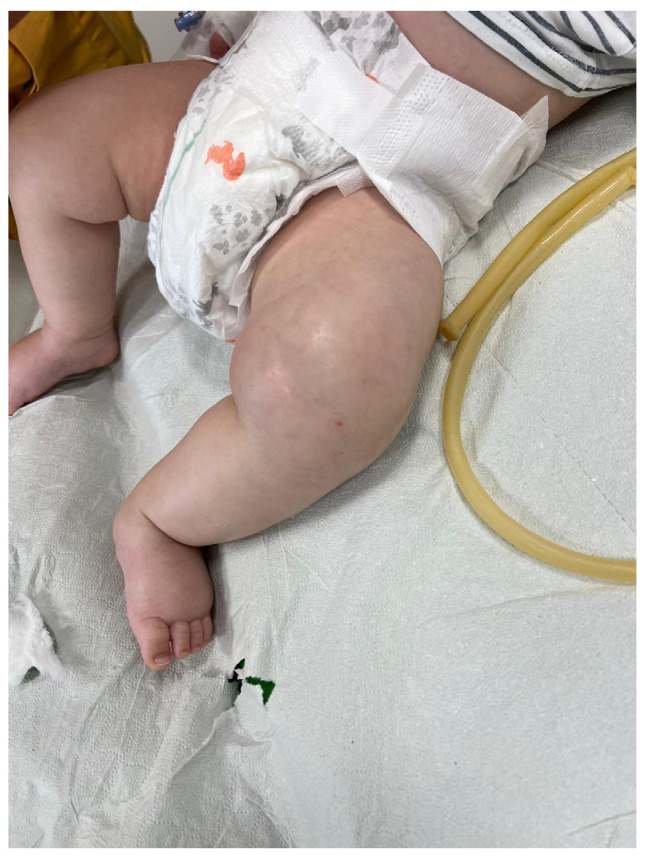
Edematous left lower limb with tense skin in a 3-month-old infant affected by osteomyelitis and soft tissue infection.

The patient’s parents did not report any prior medical conditions, and the infant exhibited good adaptation to extrauterine life and regular growth. The following findings were noted upon physical examination: SpO2, 98% in room air; heart rate, 170 bpm; respiratory rate, 35 breaths/min; and temperature, 38.5 °C. The left lower limb appeared externally rotated and flexed. The supra-patellar and thigh regions were edematous, with taut, warm, and tender skin (Figure 1).

Upon admission, antibiotic therapy was initiated with daptomycin 10 mg/kg/day and meropenem at the dosage of 60 mg/kg/day, divided into three administrations. The outcomes of haemato-chemical exams performed at various times during recovery are detailed in Table 1, and an ultrasound showed a voluminous collection in the proximity of the left-femoral region measuring 39 × 40 mm, with elevation.

**Figure 2:**
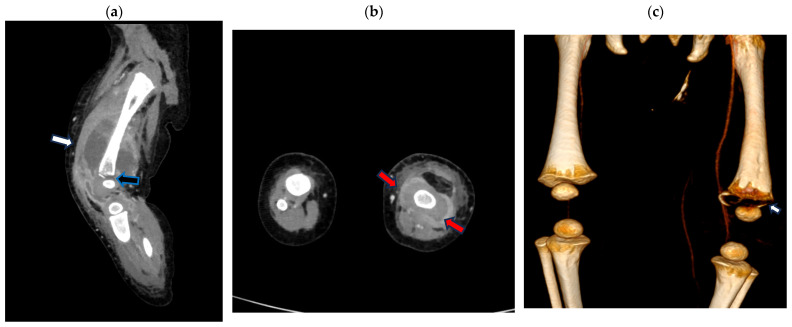
Contrast-enhanced computed tomography (CT) scan of the left leg (**a**) sagittal multi-planar (MPR) reconstruction: left fluid collection surrounding the distal metaphyseal femoral bone (white arrow) extending to the joint with the cortical erosion/destruction of the metaphyseal distal bone (black arrow); (**b**) axial MPR reconstruction: left fluid collection surrounding the distal metaphyseal femur (red arrow); (**c**) volume rendering CT reconstruction bone window demonstrating distal metaphyseal bone erosion (arrow).

A CT scan performed on the second day of hospitalization evidenced a large, encapsulated abscess collection, 9 × 4 cm in size, surrounding the distal metaphyseal femoral bone and extending to the joint. The cortical erosion of the metaphyseal distal bone, which appeared frayed, and irregularities in the ossification core were observed (Figure 2).

The surgical drainage of the abscess collection was performed, and a suction drain was placed in the left thigh. A cultural exam of the drained fluid revealed the presence of methicillin-sensitive *S. aureus* (MSSA). Therefore, antibiotic therapy was modified to cefazolin 150 mg/kg/day in three administrations for 34 days and rifampicin 10 mg/kg/day in two administrations for 32 days.

**Figure 3:**
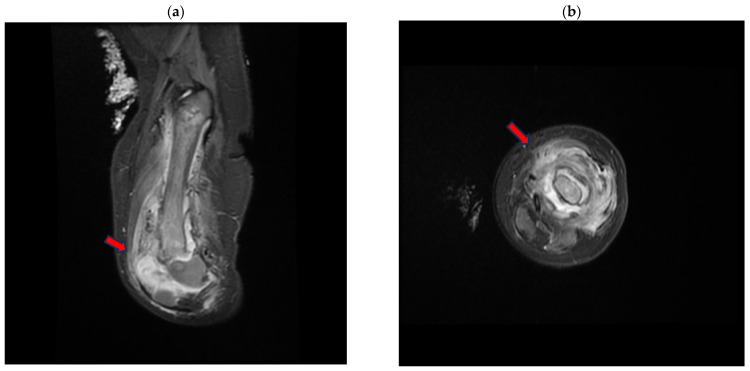
Magnetic resonance (MR) short tau inversion recovery (STIR) of the left-lower-limb coronal (**a**) and axial (**b**) planes, demonstrating the large distal metaphyseal collection (arrow) expanding in the vastus lateralis muscle with an inhomogeneous signal from the distal femoral bone.

Fifteen days after the start of specific antibiotic therapy, an MRI was performed and showed a size reduction in the peripheral collection at the left-femoral diaphysis and the distal meta-epiphyseal region (Figure 3).

The maximum thickness was approximately 9 mm in the anterior distal location, compared to the previous 16 mm.

During hospitalization, a gradual yet progressive improvement in the patient’s clinical condition was observed, with the resolution of fever and the normalization of inflammatory markers (Table 1). Signs and symptoms, including pain, improved steadily, and the active and passive mobilization of the left lower limb resumed. Three weeks after admission, the child contracted influenza A.

Four weeks of intravenous antibiotic treatment were completed, and the patient was discharged in good clinical condition after receiving a single dose of dalbavancin at the dosage of 22.5 mg/kg. Three weeks after discharge, an outpatient re-evaluation and a total body PET scan were performed, and a modest radiotracer uptake, possibly a consequence of the inflammatory process, was noted in the distal part of the left femur. Despite the steady recovery, a discrepancy in limb length was reported at follow-up.

## 3. Methodology

We searched VAERS in December 2023 for reports of osteomyelitis in children (ages 0 to 17 years) vaccinated between 1990 and 2023. The following manifestations were selected, with the corresponding codes: osteomyelitis (10031252); osteomyelitis acute (10031253); osteomyelitis bacterial (10065237); osteomyelitis chronic (10031256); and staphylococcal osteomyelitis (10064250) [23].

We included all cases where a diagnosis of osteomyelitis in childhood was clearly reported and followed a vaccination administered subcutaneously or intramuscularly.

Information gathered by VAERS is subject to reporting biases, inaccuracy, and incompleteness. Reports can be submitted by the public at large, and the numerosity of reports of events following vaccinations is not sufficient to infer causation. To counter these limitations, we excluded layperson reports, reports where data were insufficient to determine a diagnosis of osteomyelitis, and cases where a correlation between the vaccination procedure and osteomyelitis was not plausible, for instance, because of the vaccination route, the timing of symptom onset, or the occurrence of a trauma that better explained the infection. Data collected from this resource should be interpreted within the context of the existing literature.

## 4. Results

The search returned 113 total events. Sixty-six were excluded for various reasons. In 32 cases, a diagnosis of osteomyelitis was not confirmed; in 11 cases, osteomyelitis was related to the Bacillus of Calmette–Guérin (BCG) vaccine; 17 cases were excluded because a correlation with the vaccination procedure was unlikely due to the vaccination route (i.e., oral vaccines), the identified pathogens (Pneumococcus in ten cases, Mycobacterium Avium Complex in one case, and Hemophilus in one case) or the timing of symptom onset; and 6 reports were excluded because they were duplicates or not submitted by health professionals. A total of 47 cases of pediatric osteomyelitis following vaccination were selected (detailed in Table 2).

Twenty-four were female (52%). The mean age was approximately 4.7 years, with an interquartile range (IQR) between 0.85 and 10.5, and the median age was 2 years. Three were newborns (6%), 66% were up to 4 years old (*n* = 31), 6% were between 5 and 9 years old (*n* = 3), and 28% were between 10 and 17 years old (*n* = 13).

In 77% of the cases where this information was available, osteomyelitis affected the same limb into which the vaccine was injected. The time elapsed between vaccination and symptom onset was reported in all cases but five. The mean time to onset was 4.3 days (IQR, 1.0–5.7; range, between 0 and 23 days). The median time to onset was 2 days. In 86% of cases where the time to onset was known (*n* = 36), symptoms manifested within a week. Only in 7% of cases (*n* = 3) did more than 15 days pass before the onset of clinical manifestations (19, 20, and 23 days).

Ultrasound findings included soft-tissue edema and fluid collections near the infection site or underneath the periosteum. X-ray findings included new bone formation, alterations in bone density, and pathological fractures. MRI was the most frequently reported imaging technique, and findings included subperiosteal or soft-tissue fluid collections (indicative of abscesses or phlegmons), soft-tissue edema and the involvement of muscles and joints (myositis or arthritis), and periosteal reaction or elevation. In four instances, scintigraphy findings of hypercaptation indicative of increased metabolic activity were reported.

In 56% of cases (13 out of 23) where an etiological diagnosis was reported, *S. aureus* species were isolated, either from a wound or blood sample. In two cases, Group A *Streptococcus pyogenes* was isolated from the blood; in one case, *Citrobacter freundii*; in another case, it was unspecified “Gram-positive cocci”; and in another case, *S. capitis*, *S. epidermidis*, and *Pseudomonas aeruginosa* were isolated together with *S. aureus*. In six cases, cultures were negative. Blood samples were used for cultures in all cases but one, in which a wound sample was used.

Information on antibiotic sensitivity was reported only in seven of these cases: in four, MRSA was isolated, and in three, MSSA was isolated.

Treatment included antibiotics and the incision and drainage of abscesses.

In several cases, reports included information on long-term sequelae, such as permanent disability, persistent unstable gait, limb length discrepancy, bone deformities, and impaired bone growth.

The outcome was positive in 25 cases out of 30 where information on recovery was available. In the remaining five cases, the children had not yet recovered at the time that the report was submitted.

## 5. Discussion

Vaccines are crucial in the prevention of infectious diseases and have saved millions of lives over the course of the last century [1]. Immunization side effects are infrequent and are mild in most cases [24]. Although rare, infections following intramuscular or subcutaneous injections, including vaccines, can occur due to the direct inoculation of skin flora bacteria. Skin bacteria that might enter the body through injection are nearly always non-pathogenic [25,26]; however, the possibility of severe infections, especially in children and immunocompromised individuals, warrants consideration, as it can inform preventive measures.

A literature search revealed that a case of femoral osteomyelitis occurred in a newborn with no known infective risk factors one day after Hepatitis B vaccination [18]. The authors attributed the infection to inadequate site preparation and excessive depth of needle insertion [18]. About a decade later, a case of osteomyelitis localized in the arm was reported in a 15-year-old girl following HPV vaccination [17]. In this case, the infection was linked to the overpenetration of the needle during injection [17]. Reports of six similar cases of osteomyelitis in adolescents (five girls and one boy) following HPV vaccination were gathered from VAERS (Table 2). Most recently, a case of osteomyelitis caused by *C. freundii* following immunization with the Moderna COVID-19 vaccine was reported [19]. The patient, a 15-year-old boy, also developed deep vein thrombosis (DVT), a pulmonary embolism, and acute respiratory distress syndrome. The authors noted that both the vaccine itself and the bone infection could explain the thrombotic event [19].

Of the cases collected in this review, the median time to the onset of symptoms (such as fever, pain, and functional impairment) was 2 days, and in most cases (77%), the infection affected the same limb into which the vaccine was injected. Similarly, in the case report, the affected limb was the same as that of vaccination, and signs and symptoms manifested within two weeks, indicating a possible link to the injection procedure.

According to the World Health Organization (WHO), a safe injection is one that does not cause harm to the recipient, does not produce dangerous waste, and does not put the provider at risk [27]. Unsafe injections can cause viral, bacterial, or fungal infections, and recommended safety practices include hand hygiene, using gloves when appropriate or other single-use protective materials, and skin preparation through disinfection [27].

The recommended skin disinfection procedure, while varying based on the type of injection, generally consists of the use of a swab soaked with a 60–70% alcohol solution to wipe the injection site area from the center outwards [27]. The solution should be applied for 30 s, and the site should be allowed to dry for an additional 30 s [27]. Of note, according to WHO recommendations, skin preparation for vaccines should be performed with soap and water rather than with alcohol solutions [27]. According to the CDC general best practice guidelines for immunization, health practitioners administering vaccines should follow general precautions to lower the risk of infections, such as hand cleansing with an alcohol-based antiseptic or hand washing with soap and water before the procedure [28].

A review conducted by the Canadian Agency for Drugs and Technologies in Health concluded that the question of whether skin disinfection prior to injections should be performed is difficult to answer [29]. However, the criteria for inclusion in this review were met by only one randomized controlled trial (RCT) [30], where no statistically significant difference was found in infection incidence between the group of children where swabbing at the injection site was performed prior to vaccination and the control group, where alcohol swabbing was performed adjacent to the vaccination site. However, the RCT did not have sufficient statistical power to accurately assess its main outcome, skin infection, as there were no cases of pus leaking from the injection site, cellulitis, or abscesses. Additional RCTs, with a broadened focus on various types of injections, would be needed to assess the effectiveness of skin disinfection [29,30].

Several authors have questioned the utility and necessity of skin disinfection prior to injections over time, highlighting limitations in related studies, such as small sample sizes and recall bias [31,32,33,34]. A recent review on the importance of skin disinfection prior to vaccination found that only 421 out of 1534 infectious events following injections (27.4%) were due to exogenous contamination, demonstrating the role of endogenous contamination as an infective source during vaccination [35].

The Advisory Committee on Immunization Practices (ACIP) specifies that vaccines should be injected into areas where tissue injury at the neural, vascular, or cutaneous level is unlikely. Furthermore, needle length should be established based on the age and body mass of the patient, in order to reach the muscle mass while leaving nerves, blood vessels, and bone untouched [36,37,38,39]. For newborns, a 5/8” needle (22–25 gauge) is indicated, while for infants, (1–12 months) a 1” needle (22–25 gauge) is recommended [40,41]. The recommended injection site is the anterolateral thigh muscle [40,41]. Regarding the reported case, we lack details on the thoroughness of disinfection, penetration depth, or needle length used. However, inflammatory manifestations at the injection site, in the absence of a history of trauma or other infection sources, and the temporal correlation support the possibility of causality in this case.

Children are more vulnerable to osteomyelitis due to their highly vascularized, developing skeletal system combined with an immature immune system, and they face a higher risk of sequelae such as bone asymmetry and gait disturbances. In the reported case, a previously healthy child with no risk factors developed limb asymmetry due to femoral osteomyelitis following a simple preventive procedure.

Osteomyelitis is an inflammatory process with accompanied bone destruction, with long bones (like the femur and tibia) being the most commonly affected sites in pediatric age [42]. The infection can spread to the adjacent joint space in areas where the metaphysis is intracapsular, such as the proximal ends of the femur, humerus, and tibia. Bone infection can develop due to the hematogenous dissemination, contiguous spread, or direct inoculation of bacteria [43], and, in children, it typically follows trauma, which acts as an infective source leading to bacteremia and bone infection [42]. In our case, we hypothesize that the infection was caused by either direct inoculation in the bone tissue due to excessive needle penetration or the hematogenous dissemination of cutaneous bacteria.

The pathogen most commonly responsible for osteomyelitis is *S. aureus*, a frequent colonizer of human skin and mucous membranes [44,45], which was responsible for most of the cases of osteomyelitis that we reviewed with a known etiological diagnosis. In our case report, MSSA was isolated from drained fluid. *S. aureus* possesses surface receptors known as MSCRAMMs, microbial surface components that bind to adhesive matrix molecules, mediating its adhesion to bone matrix components [42,46]. Once attached to the bone tissue, *S. aureus* survives in the intracellular space favored by its metabolically quiescent phenotype [47], which makes its eradication through antibiotics challenging.

Other bacteria responsible for pediatric osteomyelitis are *S. pyogenes*, *S. pneumoniae*, Salmonella, *H. influenzae*, and *K. kingae* [43,44]. Among the reviewed cases, other isolated pathogens besides *S. aureus* included *S. pyogenes*, *S. capitis*, *S. epidermidis*, and *P. aeruginosa*. These microorganisms can be part of the skin flora, reinforcing a possible causal link with the injection procedure.

Acute osteomyelitis is more common in children, particularly under 16, than adults [48,49,50]. In children, the rich vascularization and the stagnant blood in venous sinusoids, especially at the distal ends of long-bone metaphyses, can favor the establishment of microorganisms when bacteremia occurs [51]. Bone microtrauma can also favor infection, as it affects local blood supply and causes the exposure of host matrix proteins to which bacteria can adhere.

The treatment of acute osteomyelitis in children includes an antibiotic course over 4–6 weeks [52], pain medications, and sometimes surgical procedures. In our case, empirical intravenous treatment was started and later rationalized based on the cultural exam.

Regarding the minimum duration of oral treatment, the current data are insufficient to establish a clear recommendation. We decided on a long-course intravenous treatment given the young age of the patient and the severity of the clinical picture. We opted for a 4-week treatment followed by one dose of dalbavancin, instead of a 6-week-long treatment, to reduce the length of hospitalization and its associated risks. The use of dalbavancin, approved in the pediatric population to treat acute bacterial skin and skin structure infections (ABSSSIs), has the advantage of single-dose administration, with subsequent pain reduction and early discharge, minimizing the risk of nosocomial infections and hospitalization costs [53].

In conclusion, the necessity of skin preparation prior to injection is debated, and more blinded RCTs would be necessary to remove doubts on this matter. However, to minimize the risks, costs, and potentially severe sequelae of infections related to the immunization procedure, it is important to adhere to appropriate healthcare practices concerning vaccinations. This includes the use of single-use materials, the selection of the correct needle length and site, and proper skin preparation.

## Figures and Tables

**Table 1:** Results of the haemato-chemical laboratory tests performed at different times during the hospital stay.

Variable	Upon Admission	During Hospitalization *	Upon Discharge	Reference Ranges
White blood cells (U/L)	24.520	10.680	4.020	7.90–13.40
Neutrophils	65.4%	10.6%	6.1%	17.0–55.5
Lymphocytes	24.6%	64.6%	75.2%	16.0–68.0
Monocytes	8.9%	9.2%	15.1%	4.0–11.0
Platelets (U/L)	603.300	335.400	133.100	215.0–448.0
PCR mg/dL	26.06	1.40	0.17	0.00–0.50
PCT μg/L	0.40	0.04	0.04	<0.5

* Roughly one month after admission.

**Table 2:** Summary of the reports included, collected from VAERS.

Sex/Age	Year Vaccinated *	Vaccine and Site	Days to Onset	Clinical Picture and Diagnosis	Imaging Investigation Reports	Identified Pathogens and Culture Results	Reported Treatments	Recovered at the Time of Report Submission †
M/15	2023 *	COVID-19/RA MODERNA	3	Pain, locally increased temperature, and pitting edema in the right leg extending up to the right thigh; “gray-blackish discoloration” of the affected leg; difficulty walking. Chronic osteomyelitis of the right tibia complicated by fistulization and septic arthritis in the right knee and ankle. Additional events including DVT, pulmonary embolism, ARDS, and pneumothorax	US of the leg showed DVT	Citrobacter freundii (culture of a sample from the wound)	Piperacillin/tazobactam, linezolid and clindamycin. Emergency debridement and decompression; arthrotomy, proximal tibial corticectomy, long leg casting and external fixation	No
M/12	2021	COVID-19/LA PFIZER-BIONTECH	2	Right-tibial osteomyelitis	Signal abnormality throughout the shaft of the tibia compatible with osteomyelitis; circumferential subperiosteal fluid resembling subperiosteal phlegmon or abscess; small osteochondral defect of the talus (MRI)	NR	Unspecified AB	NR
F/12	2021	COVID-19/LA PFIZER-BIONTECH	0	Fever, pain in the left buttocks and exertional pain in the left lower limb. Osteomyelitis of the left ilium, left-sacroiliac arthritis	MRI findings supported the diagnosis of osteomyelitis of the left ilium, left-sacroiliac arthritis	Negative blood culture (twice)	Cefazolin (IV, then oral)	NR
F/15	2020 *	HPV/LA (GARDASIL)	0	Severe pain in her arm and tingling in her fingers seconds after receiving the injection; the pain persisted and led to a diagnosis of frozen shoulder; persisting tenderness, swelling, and limited ROM. Acute osteomyelitis of the humerus	Osteomyelitis of the left proximal humeral metaphysis with periosteal elevation, adjacent phlegmon, myofascitis of the deltoid and triceps (MRI). Periosteal bone formation along the left proximal humeral metadiaphysis, with adjacent stippled density (heterotrophic bone formation) (XR)	Negative wound sample cultures	Cefazolin; cephalexin (oral). Incision and drainage of the abscess	Yes
M/0.08	2020	HEPATITIS B/LL (RECOMBIVAX HB)	1	Decreased movement and tenderness in the left leg. Osteomyelitis of the left tibia	Osteomyelitis of left tibia (MRI)	Negative blood culture	Ceftriaxone and clindamycin	Yes
F/5	2020	INFLUENZA/NR (QUADRIVAL A-B); PNEUMO/NR (PNEUMOVAX -23V)	1	Injection-site pain, fever, and shoulder pain. Cellulitis, osteomyelitis	Marked soft-tissue edema underlying the injection site, involving musculature, and extending to the bone near the deltoid tuberosity; periosteal thickening; and subperiosteal collection (US)	NR	NR	Yes
F/2	2019	HEP A/RL (HAVRIX)	1	Lower extremity swelling and fever. Admitted for DVT, the patient was diagnosed with MRSA bacteremia, osteomyelitis, and abscesses	NR	MRSA (blood culture)	Unspecified IV AB	Yes
M/1.33	2019	HEP A/RL. (HAVRIX); INFLUENZA/LL (FLULAVAL QUADRIVALENT); (MMR II)/LL; VARICELLA/RL (VARIVAX)	1	Pain when bearing weight on the right leg and difficulty walking, fever. Myositis, osteomyelitis, and a soft-tissue abscess in the right thigh	T2 hyperintense and diffusion restriction in the muscles, indicating myositis, and the femur, indicating osteomyelitis. A rim-enhancing fluid collection identified as a soft-tissue abscess (MRI)	NR	Unspecified AB	NR
F/1.33	2018	MEASLES + MUMPS + RUBELLA + VARICELLA/NR (PROQUAD)	20	Fever; significant claudication and difficulty bearing weight on the right leg. Right distal femur osteomyelitis	Increased uptake in the right distal femur (Scintigraphy)	Group A streptococcus pyogenes (blood culture)	Ceftriaxone IV; Cefadroxil; Cephalexin	Yes
F/1	2018	MEASLES + MUMPS + RUBELLA + VARICELLA/LA (PROQUAD)	7	Swelling and tenderness to touch in the left arm, mainly in the wrist; fever; refusal to use the left arm. Clinical diagnosis of synovial arthritis and osteomyelitis	Irregularity of cortex in left distal arm, suspicion of small fracture (XR). Increased uptake in the wrist on either side of the joint, more prominent in the growth plate of the distal left forearm (Scintigraphy)	NR	Cefuroxime; cefazolin; cephalexin	NR
M/0.17	2018	POLIO VIRUS, INACT./NR (unknown brand name and manufacturer)	1	Fever, vaccination site swelling and induration. Sepsis; right-proximal-tibia osteomyelitis; right-shoulder suppurative arthritis	NR	NR	NR	Yes
F/1	2017	HIB/LL (PEDVAXHIB); PNEUMO/LL (PREVNAR13); HEP A/RL (HAVRIX); MEASLES + MUMPS + RUBELLA + VARICELLA/RL (PROQUAD)	NR	Osteomyelitis with subperiosteal abscess in the right distal thigh	NR	MRSA (blood culture and abscess sample culture)	NR	NR
M/0.3	2016 *	HIB/NR (PEDVAXHIB); POLIO VIRUS, INACT./NR (IPOL)	NR	Fever, pain, “myositis of left lower thigh and mild periosteal reaction of left mid distal femur.”	“Myositis of left lower thigh and mild periosteal reaction of left mid distal femur” (MRI)	NR	NR	NR
F/4	2016 *	DTAP/RL (INFANRIX); VARICELLA/RL (VARIVAX)	NR	Fever and pain in the right leg. Osteomyelitis	NR	Staphylococcus (surgical sample culture)	Surgical drainage of bone infectious focus	NR
M/2	2016 *	HIB/NR; PNEUMO/NR (unknown brand name and manufacturer)	NR	History of chronic granulomatous disease; osteomyelitis	NR	NR	NR	NR
M/0.3	2016	DTP + IPV/RA (unknown brand name and manufacturer); HIB/LA (ACTHIB); HEP B/RA (unknown brand name); PNEUMO/NR (PREVNAR13); ROTAVIRUS/MO (ROTARIX)	6	Fever, cellulitis of the right upper arm, myositis, and osteomyelitis of the right radius	Osteomyelitis of the right radius (MRI). Radiolucent line near the right radius (XR)	MSSA (blood culture)	Cefotaxime; cefazolin	NR
F/1.5	2016	DTAP/LL (DAPTACEL); INFLUENZA (SEASONAL)/LL (FLUZONE QUADRIVALENT); POLIO VIRUS, INACT./LL (IPOL)	1	Bilateral hip pain; refusal to walk; fever; and decreased appetite. Right-iliac-bone early osteomyelitis	Right-sacroiliac-joint effusion, adjacent inflammatory changes, and right-iliac-bone early osteomyelitis (MRI)	NR	NR	Yes
F/17	2014 *	HPV/NR (CERVARIX)	NR	Hospitalized with a diagnosis of osteomyelitis	NR	NR	NR	Yes
M/2	2014 *	DTAP/LL (DAPTACEL); HIB/LA (PEDVAXHIB); HEP A/LL (VAQTA); MEASLES + MUMPS + RUBELLA + VARICELLA/LL (PROQUAD); PNEUMO/RA (PREVNAR13)	3	Swelling and erythema. Hospitalized with diffuse cellulitis	“Multiloculated abscesses of the humeral shaft, osteomyelitis of the distal humerus and osteomyelitis of proximal radius and ulna” (MRI)	Gram+ cocci (blood culture)	Unspecified surgical treatment	NR
F/5	2013	DTAP/LL; POLIO VIRUS, INACT/LL unknown brand name and manufacturer	1	Unable to bear weight on the right ankle. Diagnosed with osteomyelitis localized in the right ankle	NR	NR	Unspecified IV AB	No
M/2.7	2012	DTAP/LL (INFANRIX); POLIO VIRUS, ORAL, unknown brand name and manufacturer	1	Fever, erythema at the injection site, hip edema. Hospitalized with a diagnosis of hematogenic osteomyelitis	NR	NR	Unspecified surgical treatment	No
M/4	2012	HIB/LL (PEDVAXHIB); PNEUMO/RL (PREVNAR13)	1	History of allergic rhinitis and croup; fever, left-thigh soreness and claudication; and osteomyelitis of the left femur	Left-femur osteomyelitis (MRI)	NR	Unspecified IV AB	Yes
M/13	2012	HPV/LA (GARDASIL)	13	Pain in the left arm and persistent headache. Hospitalized with bacteremia and osteomyelitis in the left humerus	Left-humeral osteomyelitis (MRI)	Staphylococcus (blood)	Cephazolin (IV), cephalexin (oral, at home)	NR
M/0.11	2012	DTAP + IPV + HIB/LL (PENTACEL); PNEUMO/LL (PREVNAR13); ROTAVIRUS/MO (ROTATEQ)	0	Edema of the thigh and inability to move the leg. Left-femur osteomyelitis	Periosteal elevation (XR), muscle abscess (MRI)	MSSA (abscess sample culture)	Unspecified IV AB	Yes
F/0.1	2011 *	HEP B/LL (RECOMBIVAX HB)	7	Erythema and edema in the right inferolateral thigh, reduced ROM, and fever	Abscess (US), periosteal reaction in the femur, coherent with osteomyelitis (XR)	Gram+ cocci (microscopy) and MRSA (blood and abscess culture)	Teicoplanin, cefotaxime. Abscess drainage and debridement	NR
M/4	2011	PNEUMO (PREVNAR13)	0	Local reaction, claudication involving the left leg, malaise and aches, fever, and pain in the right elbow. “Hospitalized for osteomyelitis”, significant improvement after AB treatment	Negative MRI	Negative blood culture	Unspecified AB	Yes
F/0.42	2010	DTAP/NR (INFANRIX); PNEUMO/NR (SYNFLORIX)	0	Fever and hesitancy to bear weight on the right leg. Diagnosis of osteomyelitis	Negative XR and US	Negative blood culture	Cefotaxime (IV), flucloxacillin (oral)	NR
F/0.02	2009	HEP B (FOREIGN)	4	Maternal fever of unknown origin 4 days before delivery; decreased movement and pain on extension of the left lower leg, pain in the left hip, swollen left ankle, and fever. Septic left hip and presumed osteomyelitis in the left ankle	Leg XR and hip US negative; radiological diagnosis of ankle osteomyelitis “pending” at the time of the report	Methicillin-sensitive *S. aureus* (MSSA) (blood culture, ankle abscess sample)	IV oxacillin	Yes
F/12	2008	HPV/RA (GARDASIL)	0	Osteomyelitis of left arm	NR	NR	NR	No
F/12	2008	DT + IPV/NR (unknown brand name and manufacturer); HPV/RA (GARDASIL)	7	Arthralgia of left ankle, with edema and erythema; fever; and “osteomyelitis of septic genesis”	XR of ankle was normal	NR	Unspecified AB	Yes
F/11	2008	HPV/NR (GARDASIL)	0	Complaints of swelling; patient was later hospitalized and diagnosed with osteomyelitis	NR	NR	Unspecified AB	Yes
M/4	2008	MMR II/NR	4	Osteomyelitis	NR	NR	NR	No
F/11	2007	PNEUMO/RA (PNEUMOVAX)	2	Erythema, tenderness, induration at the sight of the injection; fever, malaise, and generalized weakness; decreased ROM; and cellulitis and osteomyelitis in the upper right arm Suspected underlying immunodeficiency	Cellulitis and osteomyelitis of the right proximal humerus (MRI)	NR	Unspecified AB	Yes
M/1.02	2007	HIB + HEP B/LL (COMVAX); MMR+VARICELLA/LL (PROQUAD)	15	Fever, refusal to walk, irritability; left-pelvic myositis and fasciitis; osteomyelitis of the left ileum and sacrum	Septic arthritis in the left sacroiliac joint, osteomyelitis of the left ileum and sacrum, pyomyositis of the adjacent musculature with an intramuscular abscess (MRI). Fluid in right hip joint space (US)	NR	IV AB, oral clindamycin. Muscle abscess drainage, septic joint drainage	Yes
M/8	2005	INFLUENZA/NR (SEASONAL) (FLUMIST)	4	Fever, vomiting, pain in the right leg, foot, wrist, and elbow, inability to walk, and group A streptococcal bacteremia, osteomyelitis, and arthritis	NR	Group A streptococcus (blood)	Unspecified AB	NR
F/0.34	2005	DTAP/NR (INFANRIX); HIB/NR (HIBERIX); POLIO VIRUS, INACT./NR, unknown brand name and manufacturer	5	Osteomyelitis in the ankles	NR	NR	Amoxicillin trihydrate + potassium clavulanate	NR
F/0.28	2005	DTAP + HEPB + IPV/LL (PEDIARIX); HIB/RL (ACTHIB); PNEUMO/RL (PREVNAR)	9	Fever, irritability, abdominal pain; bilateral otitis media; swelling of left upper leg; osteomyelitis of left femur and fifth-digit abscess. Chronic osteomyelitis of the left femur	Increased density of soft tissue due to edema, the stranding of subcutaneous tissue, and left quadriceps musculature; “linear metaphyseal lucency of distal femur” (XR). Subperiosteal abscess in the left femur, antero-lateral in the proximal region and circumferential in the distal portion (CT). Pathological fracture of the proximal portion of the femoral metaphysis (XR).	MRSA and coagulase-positive Staphylococcus (culture of wound samples from leg and finger)	IV clindamycin and IV vancomycin and rifampicin. “Ultrasound guided incision and extensive drainage of subperiosteal abscess of left femur; drainage of fifth digit abscess; Surgery of a large subperiosteal abscess (recurrence) and a small intramuscular abscess”	Yes
F/1	2004	MMR II/NR	7	Fever, trismus, painful swelling of the right jaw and cervical lymphadenopathy; history of teething; and acute osteomyelitis of the jaw	Large soft-tissue mass, improved after treatment, osteolytic foci and pathological fracture in the right jaw, the loss of local fat planes, and the infiltration of subcutaneous fat (CT)	NR sterile blood culture	Amoxicillin–clavulanic acid	NR
M/0.08	2003	DTP + HEP B/LL (TRITANRIX)	3	Fever, induration, and swelling at the injection site, thick purulent collection, and osteomyelitis of the femur	NR	NR	Ceftriaxone	NR
F/4	2003	DTAP/RL (INFANRIX); MMRII/LL; IPOL/LL; VARICELLA/RL (VARIVAX)	23	Fever, extreme pain in knee joint. Osteomyelitis in the right femur	NR	*S. aureus* (surgical sample)	Cefazoline. Surgical drainage of infective focus	Yes
M/1.6	2002	DTAP/LL (INFANRIX); HIB/RL (ACTHIB); MMRII/RL; VARICELLA/RL (VARIVAX)	0	Fever, irritability, reduced sleep and eating, and seizures. Later report of *S. aureus* osteomyelitis, pyomyositis, anemia, and leukopenia	NR	*S. aureus* (sample NR)	NR	Yes
M/0.7	2002	DTAP/NR (TRIPEDIA); HIB/NR (ACTHIB); POLIO VIRUS, INACT./NR (IPOL)	4	Acute osteomyelitis caused by the same pathogens (not specified) isolated from his throat	NR	NR	Unspecified AB	Yes
F/1	2001	MMR II/RL	0	Injection site hematoma and local induration persisting for 3–4 months, evolving over the course of more than a year in an abscess with fistulization and lymphadenopathy. The abscess recurred a first time after surgical treatment and scarring and a second time a month after medical treatment. A physician concluded that this was due to chronic osteomyelitis with osteolysis of the right iliac bone and significant fistulization	Lesion with fluid compartments at the injection site (US); an MRI of the pelvis showed muscular infection and damage to the right iliac wing and confirmed the extension of the infective focus	*S. aureus*, *S. capitis*, *S. epidermidis* and *Pseudomonas aeruginosa* were isolated at different times from samples taken from the wound	Erythromycin and pristinamycine, oracilline and neomycin, and cotrimoxazole. Necrosectomy and curettage of the soft tissue	Yes
M/10	1999	HEP B/LA (ENGERIX-B)	2	Unable to walk due to acute left-gluteal pain. Diagnosed with osteomyelitis and abscess located in the left ilium	XR aided the diagnosis of osteomyelitis and abscess	NR	NR	Yes
M/11	1998	HEP B/NR (RECOMBIVAX HB)	0	Fever and pain in the right leg. Diagnosed with osteomyelitis of the right leg	NR	NR	NR	Yes
F/11	1996	HEP B/NR (ENGERIX-B)	19	Fever, shoulder, elbow, ankle and knee arthralgia; patchy erythema and vasculitis; osteomyelitis dx	Hypercaptation on the right (bone scintigraphy)	NR	NR	Yes
NR/1	1994	HEP B/LL (RECOMBIVAX HB)	2	Febrile illness, inability to use the left leg for 5 days. Diagnosed with osteomyelitis of the left femur	NR	*S. aureus* (blood culture)	NR	Yes

Acronyms and abbreviations: LL (left leg), LA (left arm), RL (right leg), RA (right arm), HEP B (hepatitis B vaccine), DTAP (Diphtheria and Tetanus Toxoids and Acellular Pertussis Vaccine), HIB (Haemophilus B Conjugate Vaccine), PNEUMO (Pneumococcal Vaccine), POLIO VIRUS (poliovirus vaccine inactivated), DTP + HIB (Diphtheria and Tetanus Toxoids Pertussis and Haemophilus Influenza B Vaccine—Hexavax), MMR II (Measles, Mumps, and Rubella Virus Vaccine, Live), INACT. (inactivated), IPOL (poliovirus vaccine, inactivated), TDAP (ADACEL) (Tetanus and Diphtheria Toxoids and Acellular Pertussis Vaccine—Boostrix/Adacel), ROM (range of motion), DT + IPV (DT-IPV Combined DT and IPV Vaccine), DTAP + HEPB + IPV (Diphtheria and Tetanus Toxoids and Acellular Pertussis Vaccine + Hepatitis B + inactivated poliovirus vaccine), XR (X-ray), MRI (magnetic resonance imaging), CT (computerized tomography), MO (mouth), AB (antibiotics), NR (not reported), MRSA (Methicillin-resistant *Staphylococcus aureus*), MSSA (Methicillin-sensitive *Staphylococcus aureus*). * When the year of vaccination was missing, we reported the year in which the report was submitted, which often coincided with the vaccination year. † This indicates the recovery status at the time that the report was submitted to VAERS. “Yes” indicates that the child had already shown clinical improvement or had been discharged form the hospital, “No” indicates that the child had not yet recovered at the time of report submission.

## Data Availability

All relevant data are included in the article.

## Abbreviations

AB	Antibiotics
ABSSSI	Acute bacterial skin and skin structure infections
ACIP	Advisory Committee on Immunization Practices
AIFA	Agenzia Italiana del Farmaco (Italian Medicine Agency)
ARDS	Acute respiratory distress syndrome
CDC	Centers for Disease Control and Prevention
CT	Computed tomography
DTaP	Diphtheria, Tetanus, and Pertussis Vaccine
DT-IPV	Diphtheria and Tetanus Toxoids and Inactivated Poliovirus Vaccine
DVT	Deep vein thrombosis
Hep B	Hepatitis B vaccine
Hib	Haemophilus influenzae type b vaccine
HPV	Human papillomavirus vaccine
IPV	Inactivated poliovirus vaccine
LA	Left arm
LL	Left leg
MMR	Measles, Mumps, and Rubella Vaccine
MO	Mouth (in the context of vaccine administration)
MPR	Multi-planar reconstruction
MRSA	Methicillin-resistant *Staphylococcus aureus*
MRI	Magnetic resonance imaging
MSSA	Methicillin-sensitive *Staphylococcus aureus*
NR	Not reported
Pneumo	Pneumococcal vaccine
RA	Right arm
RCT	Randomized controlled trial
RL	Right leg
RNF	Rete Nazionale di Farmacovigilanza (National Pharmacovigilance Network)
ROM	Range of motion
STIR	Short tau inversion recovery
Tdap (Adacel, Boostrix)	Tetanus, Diphtheria, and Pertussis Vaccine (specific brands)
US	Ultrasound
VAERS	Vaccine Adverse Event Reporting System
WHO	World Health Organization
XR	X-ray
